# Extrapulmonary Tuberculosis Leading to Abdominal Wall Mass in Young Patient

**DOI:** 10.1155/2024/9924307

**Published:** 2024-02-05

**Authors:** Mohadeseh Karimi, Ali AtashAbParvar

**Affiliations:** Department of Pathology Medical Sciences, University of Hormozgan, Bandar Abbas, Iran

## Abstract

**Background:**

Tuberculosis is a bacterial infection that is caused by *Mycobacterium tuberculosis*. Tuberculosis has arguably been the largest killer of humans historically, and it remains one of the most important infectious causes of death in the world. Tuberculosis can be classified into different forms and it manifests as pulmonary and out pulmonary, respectively, in 85% and 15% of cases. Only a few cases of tuberculosis with abdominal wall involvement have been reported. *Case Presentation*. Herein, we present a 27-year-old Persian woman, presented with asymmetric abdominal bulging in the right side of the periumbilical area since 6 months before admission that has no pain or secretion. The patient was oriented and not ill or toxic in general appearance. Vital signs were within normal ranges. An abdominal physical examination revealed a mobile, well-bordered, nontender mass 3 × 3 centimeter (cm) in diameter palpated in the right periumbilical area. The patient underwent surgery which revealed a cystic lesion that was carefully resected. In histopathology examination of the specimen resection, tuberculosis was confirmed.

**Conclusion:**

We report a rare case of extrapulmonary tuberculosis that was identified at the abdominal wall. Due to the high number of cases of TB and the development of extrapulmonary forms that can present in an unusual location as an abdominal wall which are difficult to diagnose, it is very important to keep in mind the differential diagnosis of tuberculosis.

## 1. Introduction

Tuberculosis is a bacterial infection that is caused by *Mycobacterium tuberculosis* [1].

Tuberculosis has arguably been the largest killer of humans historically, and it remains one of the most important infectious causes of death in the world. The incidence of tuberculosis and mortality has been declining during the past decade, despite the global burden remaining large, with over 10 million people per year newly ill with the disease [2]. Although tuberculosis is a preventable and curable disease, 1.2 million people died of TB among HIV seronegative patients in 2019 [3].

Tuberculosis can be classified into different forms, and it manifests as pulmonary and out pulmonary, respectively, in 85% and 15% of cases [4]. These various manifestations of tuberculosis are seen in developing countries that depend on mycobacterium virulence and host immune status. Musculoskeletal tuberculosis occurs in 1–3% of patients with tuberculosis [5, 6]. The involvement of abdominal wall muscles can occur by direct inoculation from caseous lymph nodes or hematogenous dissemination from the primary focus [7]. Only a few cases of tuberculosis with abdominal wall involvement have been reported [8].

According to the high prevalence of tuberculosis in the world and the rare occurrence of abdominal wall tuberculosis, we report a case of a 27-year-old female patient with tuberculosis presenting as an abdominal wall mass.

## 2. Case Presentation

A 27-year-old Persian woman presented with asymmetric abdominal bulging in the right side of the periumbilical area 6 months before admission and had no pain or secretion. There was no history of poor appetite, severe fatigue, nocturnal hyperhidrosis, dyspnea, or constipation. The patient had lost about eight kilograms during three months without changing his diet. Social history revealed that she has been smoking since 10 years ago. Family history is negative.

The patient was oriented and not ill or toxic in general appearance. Vital signs were within normal ranges. Her abdominal physical examination revealed a mobile, well-border, nontender mass 3 × 3 centimeter (cm) in diameter palpated in the right periumbilical area. Bowel sounds are normal. Shifting dullness and fluid wave tests and Murphy's sign were negative. On systemic physical examination, the patient had pale conjunctiva without yellowing of the skin or sclera. All cardiopulmonary examinations were negative; there was no spider nevus; the liver and spleen were not palpable under the ribs; there was no percussion pain in the liver and spleen; there was no vascular murmur; and all neurological investigations were normal. Laboratory tests were requested which are shown in Table 1.

**Figure 1:**
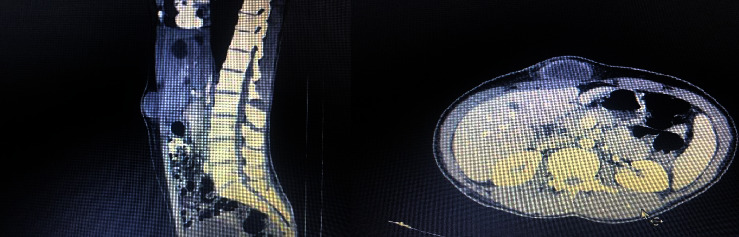
Abdominal CT scan with contrast shows a cystic lesion with wall thickness internal echogenicity and fat stranding without enhancement.

Preoperatively, the patient was assessed with an abdominal CT scan with contrast which showed a cystic lesion with wall thickness, internal echogenicity, and fat stranding without enhancement (Figure 1).

The patient underwent abdominal cyst aspiration, and 10 ml yellow fluid was received, centrifuged, and three smears prepared which was stained using the Wright‐Giemsa and Pap's methods. The slides show hypocellular smears composed of some lymphocytes distributed in a fine granular background. Cyst drainage fluid culture revealed negative. Further investigation on cyst fluid such as acid-fast bacilli (AFB) staining was negative and the adenosine deaminase (ADA) level was 34 IU/L.

**Figure 2:**
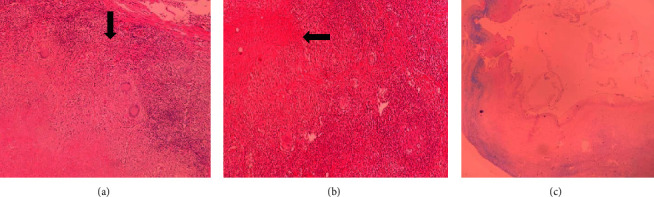
(a, b) Caseous granulomatous infection surrounded by Langerhans's multinucleated giant cells (see black arrows). (c) Acid-fast staining of the tissue biopsy was negative.

The patient underwent surgery. Intraoperatively, the skin and subcutaneous were opened from the right para midline, and the lesion was carefully resected. The incision site was repaired, and the resected specimen was sent to the pathology laboratory. The specimen was only the cyst wall measuring 40 *∗* 25 mm that was cut into multiple pieces in five blocks, and 20% of it was embedded. The histopathological examination showed fibroconnective tissue with the presence of lymphoid cells, lymphoid follicle formation with a germinal center accompanied by caseous granulomatous formation surrounded by Langerhans multinucleated giant cells that tuberculosis was confirmed in specimen resection (Figure 2). Acid-fast staining of the tissue biopsy was negative, and a real-time polymerase chain reaction (PCR) test (by roje company kit) of it was positive. The patient was kept in follow-up for about 6 months, with only vital sign checking and physical examination, without antituberculosis treatment. The patient had no complaints, and physical examination revealed normal.

## 3. Discussion

Tuberculosis is a bacterial infection that is caused by *Mycobacterium tuberculosis* [1].

Tuberculosis has arguably been the largest killer of humans historically, and it remains one of the most important infectious causes of death in the world. The incidence of tuberculosis and mortality has been declining during the past decade, despite the global burden remaining large, with over 10 million people per year newly ill with the disease [2]. Although tuberculosis is a preventable and curable disease, 1.2 million people died of TB among HIV seronegative patients in 2019 [3].

The progression of tuberculosis can be affected by socioeconomic factors and genetic or molecular changes related to the host immune response [9–11].

Furthermore, earlier diagnosis of extrapulmonary tuberculosis is important to prevent progression to the contagious state and spare individual patients' significant morbidity and mortality.

Although it primarily involves the lung, the incidence of extrapulmonary tuberculosis has been rising recently which has become the main global health issue. The most prevalent extrapulmonary site was reported to be the pleura, followed by the lymph nodes, musculoskeletal, genitourinary, abdomen, and central nervous system [12, 13].

Although most cases of tuberculosis are pulmonary tuberculosis, extrapulmonary tuberculosis leads to a large number, longer times, and higher costs of hospitalizations compared to pulmonary tuberculosis, but there was no difference in mortality. However, miliary tuberculosis and tuberculosis of the meningeal and central nervous systems increased the risk of mortality [14].

The involvement of abdominal wall muscles can occur by direct inoculation from caseous lymph nodes or hematogenous dissemination from the primary focus [7].

Only a few cases of tuberculosis with abdominal wall involvement have been reported [8]. Our patient presented with asymmetric abdominal bulging on the right side of the periumbilical area 6 months before admission and has no pain or secretion. Moreover, an abdominal CT scan with contrast showed a cystic lesion with wall thickness, internal echogenicity, and fat stranding without enhancement.

Extrapulmonary tuberculosis also has pulmonary involvement in about 10–50% of people, but the disease can occur without pulmonary symptoms as isolated forms that represent 15–20% of all tuberculosis cases [15]. Our patient had no evidence in favor of lung involvement.

The patient underwent surgery which revealed a cystic lesion that was carefully resected.

The diagnosis of extrapulmonary tuberculosis is often missed with a significantly lower diagnostic rate during the first visit than for pulmonary tuberculosis or combined tuberculosis (21.16% vs. 34.67% and 44.74%). Extrapulmonary tuberculosis often obscures detection due to the low bacterial load in nonrespiratory specimens [16]. Histopathology examination, polymerase chain reaction (PCR) test, and acid-fast bacilli (AFB) staining were used for the diagnosis of tuberculosis in our study.

## 4. Conclusion

Due to the high number of cases of TB and the development of extrapulmonary forms that can present in an unusual location, such as an abdominal wall which are difficult to diagnose, it is very important to keep in mind the differential diagnosis of tuberculosis.

## Figures and Tables

**Table 1:** Laboratory findings of the patient.

Test	Result	Reference range
WBC (10^9^/L)	8.3	4.0–11.0
Neutrophil	53.2%	
Lymphocyte	46.8%	
RBC (10^6^*/µ*L)	5.24	
RDW	15.4%	
HB (g/dL)	11.4	13–16
PLT (10^3^/*µ*L)	230	150–450
Urea (mg/dL)	32	11–55
Cr (mg/dL)	1.1	0.6–1.3
AST (U/L)	28	<37
ALT (U/L)	11	<41
ALP (U/L)	245	100–360
Bili T (mg/dL)	1.2	0.3–1.2
Bili D (mg/dL)	0.4	≤0.3
PT (sec)	12	12–14
PTT	31.2	25–45
INR (sec)	1.0	≤1.1
Sodium (mEq/L)	137.9	135–145
Potassium (mmol/L)	4.11	3.5–5

## Data Availability

The datasets used during the current study are available from the corresponding author on reasonable request.
